# The Farsi version of Caregiver Preparedness Scale in Iranian family caregivers of the older adults undergoing hemodialysis: a psychometric study

**DOI:** 10.1186/s12877-024-05103-0

**Published:** 2024-06-12

**Authors:** Arash Kian, Hamid Sharif-Nia, Sima Hejazi

**Affiliations:** 1 Student Research Committee, Department of Nursing, Bojnurd School of Nursing, North Khorasan University of Medical Sciences, Bojnurd, Iran; 2 Psychosomatic Research Center, Mazandaran University of Medical Sciences, Sari, Iran; 3 Department of Nursing, Amol Faculty of Nursing and Midwifery, Mazandaran University of Medical Sciences, Sari, Iran; 4 Department of Nursing, Bojnurd School of Nursing, North Khorasan University of Medical Sciences, Shahriar Ave, Bojnurd, Iran

**Keywords:** Psychometrics, Caregiver preparedness scale, Family Caregiver, Older adults, Chronic kidney disease, Hemodialysis

## Abstract

**Background:**

Enhancing preparedness of family caregivers and support for caregiving is essential for the mutual benefit of both caregivers and the well-being of those under their care. This study aimed to translate and evaluate psychometric properties of the Caregiver Preparedness Scale among family caregivers of older adults undergoing hemodialysis.

**Methods:**

In this methodological study, 400 family caregivers of older adult patients undergoing hemodialysis enrolled to the study via convenience sampling method. The study was conducted in two stages: translation and psychometric evaluation. At first, the translation of the scale was done using Beaton et al. method. In the psychometric evaluation stage, quantitative face validity, content validity, item analysis and construct validity of the scale were evaluated. The internal consistency of the scale was assessed through the calculation of Cronbach’s alpha, McDonald’s omega, and average inter-item correlation coefficients.

**Results:**

All items had an impact score greater than 1.5. The content validity ratio and the kappa coefficient for all items were above 0.75. In the item analysis, item 2, which had a correlation with the total score of less than 0.3, was removed. Following exploratory factor analysis, only one factor composed of all items (7 items) was extracted, explaining 75.7% of the total variance. This model had acceptable fit indices in confirmatory factor analysis. Cronbach’s alpha and omega of 0.95 and an average inter-item correlation of 0.75 were obtained.

**Conclusions:**

The study results demonstrated that the Caregiver Preparedness Scale exhibits appropriate psychometric properties. Geriatric nurses can utilize this Scale for assessment of caregivers. This assessment can aid in decision-making regarding educational programs aimed at enhancing family caregiver preparedness.

## Introduction

Chronic kidney disease is one of the prevalent chronic diseases in the older adults. Research results indicate that the age pattern of chronic kidney disease is trending towards the older adults, and over the past two decades, the number of older adult patients with this disease has been on the rise in most countries [1]. In 2017, the global prevalence of Chronic kidney disease was 9.1%, which is approximately 700 million cases [2].Chronic kidney disease progresses through a five-stage path towards end-stage kidney disease, which is referred to as the end stage of chronic kidney disease. In this stage, patients require kidney replacement therapies such as dialysis (hemodialysis or peritoneal dialysis) or kidney transplantation [3]. By the end of 2020, the number of individuals worldwide undergoing kidney replacement therapies had reached over 5.2 million patients, and it is projected that this will increase to 4.5 million by the year 2030 [4, 5]. Hemodialysis is the most common treatment method for patients with end-stage kidney disease [6]. In Iran, by 2015, over 27,000 patients received treatment in 500 hemodialysis centers [7]. According to the United States Renal Data System, the prevalence and incidence of this disease have increased in individuals over 65 years old, with the average age typically ranging from 60 to 70 years in all countries [8]. In Iran, the average age of this disease is also increasing, with some studies reporting an average age of 57–60 years [9] and others reporting 60–70 years [10].Therefore, the older adults are the largest and fastest-growing group of patients with chronic kidney disease [11].

Patients undergoing hemodialysis require caregiver support in various aspects, including transportation, shopping for appropriate food, preparing meals, adhering to a specific dietary regimen, attending medical visits, organizing necessary equipment and facilities, and managing disease symptoms [3–5, 7, 12]. Most hemodialysis patients rely on their family members for assistance in daily activities and medical care, and the role of family caregivers is significant and extensive. Additionally, providing psychological and social support to patients in dealing with the stresses associated with dialysis is often the responsibility of family caregivers [13]. Family caregivers currently play a crucial and long-term role in the care system [14]. A family caregiver is someone who provides support to a family member who is sick, older adults, or disabled, without receiving payment, and assists them with personal care, medical care, and coping with the disease [15]. Supporting a family member in need of home care is a vital and complex role that comes with new responsibilities, often leaving family caregivers ill prepared. This lack of preparedness often leads to negative effects on the caregiver’s health and well-being, such as stress, anxiety, fear, guilt, and sleep disturbances [16]. Caregivers of patients undergoing hemodialysis experience lower quality of life compared to similar age and gender groups in society [17–19] and experience high levels of caregiver burden and social isolation [3]. Therefore, considering that family caregivers are a vulnerable group, both physically, mentally, and financially, and are exposed to significant pressure, supporting and enhancing their preparedness for caregiving is essential for the mutual benefit of caregivers and the well-being of those under their care [20, 21]. Studies have shown that a sense of preparedness can affect the caregiving experience and protect family caregivers from negative consequences of caregiving [16]. Preparedness, in this context, is understood as readiness in multiple areas of caregiving, including providing physical care, offering emotional support, establishing home support services, and coping with caregiving stress [22]. It is also seen as a state or capability of predicting potential problems and finding potential solutions, requiring the development of skills and abilities [16]. Preparedness is recognized as an important factor in improving caregiver resilience, and increasing caregivers’ preparedness is crucial due to its positive impact on resilience [23]. Furthermore, recent studies suggest that preparedness for caregiving should be assessed as a continuous and ongoing process since caregivers need to be prepared for potential issues and challenges as the patient’s condition changes [24, 25]. To assess preparedness, there is a need for precise assessment tools.

Based on existing studies, one of the scales introduced for assessing the needs and preparedness of caregivers, as well as evaluating interventions to meet these needs, is the Caregiver Preparedness Scale. This self-assessment scale consists of eight items designed to measure the level of family caregiver preparedness for providing care. The preparedness assessed by this scale is defined as readiness in multiple areas of caregiving, such as providing physical care, offering emotional support, establishing home support services, and coping with caregiving stress. This scale was developed by Archbold et al., (1990) in the United States to assess the preparedness of family caregivers of frail older adults living at home, and its initial validity and reliability have been examined [16, 22]. Based on research findings, there is currently no Farsi-language version of this scale in Iran, and a similar tool in Farsi is not available for assessing the preparedness of family caregivers, who are the primary caregivers for older adults with end-stage chronic kidney disease undergoing hemodialysis. The lack of such tools in the Farsi language equates to the inability to assess caregivers’ preparedness to care for their older adults. As noted, this issue may results in increased caregiver burden and psychologic issues among caregivers. This study aims to translate and psychometrically evaluate the Caregiver Preparedness Scale to provide a suitable tool for future research on the assessment and education of preparedness among caregivers of these patients.

## Methods

### Study design

The present research is a methodological study conducted from September 2022 to Jun 2023. The study consisted of two phases (translation of the scale and psychometric evaluation of it) during which the “Caregiver Preparedness Scale” was translated, and its psychometric properties were evaluated in family caregivers of older adult patients undergoing hemodialysis in Ardabil City, Iran.

### Participants and sampling

The research population were family caregivers of older adult patients with chronic kidney disease undergoing hemodialysis in Ardabil City, Iran. Inclusion criteria included having the primary responsibility for the care of an older adult patient with chronic kidney disease undergoing hemodialysis, the patient receiving ongoing hemodialysis treatment, the patient’s age being over 60 years, and proficiency in the Farsi language.

In the pre-test phase of translation and item analysis, a sample of 30 family caregivers of old individuals undergoing hemodialysis was selected through convenience sampling. Samples from each of these stages were independent of each other. In the structural validity assessment section, according to the COSMIN Risk of Bias checklist, a sample size seven times the number of items and more than 100 for conducting factor analysis is considered appropriate [26]. In this study, using a combination of available criteria, Exploratory Factor Analysis (EFA) was performed with 200 samples, and Confirmatory Factor Analysis (CFA) was conducted with an additional 200 samples independent of EFA, selected via convenience sampling method from caregivers of old individuals undergoing hemodialysis referring to Ardabil City dialysis centers (two centers out of a total of three centers in the city). In these centers, 250 and 78 patients were above 60 years old, respectively. One of these centers was located in a private facility. Some patients had more than one (two or more) primary family caregiver. Therefore, a total of 400 primary family caregivers were enrolled in the study. For the assessment of internal consistency, samples from the exploratory factor analysis were utilized. The researcher, at each stage, obtained consent and conducted sampling by visiting the hemodialysis units based on inclusion criteria and after obtaining informed written consent.

### Caregiver preparedness scale

This scale is a self-assessment scale consisting of eight items (questions) designed to assess the family caregiver’s preparedness to provide care. The scale was originally developed by Archbold et al., (1990) in the United States and was initially validated in family caregivers of frail older adult living at home. Preparedness, as assessed by this scale, is defined as the perceived preparedness for various caregiving roles, such as providing physical care, providing emotional support, setting up supportive services at home, and coping with caregiving stress. Responses are rated on a 5-point scale ranging from zero (not at all prepared) to four (very well prepared). Lower scores indicating lower caregiver preparedness [22, 27–31].

### Data Analysis

- Translation

The Caregiver Preparedness Scale was translated using the “Cross-cultural Adaptation of Self-report Measures” guideline introduced by Beaton et al., (2000) in the following stages:

#### Preparation

Obtaining permission from the original scale developer and obtaining ethical approval for the research.

#### Initial translation

Translation of the tool by two independent translators, resulting in two Farsi versions of the instrument (T1 and T2).

#### Synthesis of translations

Synthesis of translations by a group consisting of the two translators from the previous stage and a researcher, leading to a final Farsi translation (T-1, 2).

#### Back-translation

Two other blinded translators back translated the scale to the original language (English), and the scale developer approved the back-translated version.

#### Expert Committee Review

In this stage, an expert panel comprising a methodologist, experts in the fields of gerontology and nursing, linguists, translators, and the scale developer reviewed and integrated all translated versions of the scale to prepare a final pre-test version for field-testing.

#### Pretesting

Cognitive interviews and pilot testing of the final pre-test translated version were conducted with a group of 30 family caregivers of older adult patients with chronic kidney disease undergoing hemodialysis treatment in Ardabil City, Iran. This stage ensured the accuracy of interpretation and comprehension of the items and response options by caregivers.

#### Final Version Development

In the final stage, the researcher, in collaboration with the expert panel, reviewed all reports and forms related to the adaptation process and developed a final translated version for psychometric evaluation of the Farsi version of the scale [32].

### Psychometric evaluation of the Caregiver Readiness Scale

The psychometric properties of the scale were evaluated as follows:

- Face Validity

To assess quantitative face validity, the method of calculating the item impact score was used. In this method, the opinions of 10 family caregivers of older adult patients undergoing hemodialysis, who were responsible for the direct care of the patient, were gathered. The impact score of each item was calculated, and items with an impact score less than 1.5 were decided to be retained [33, 34].

- Content Validity

In assessing content validity, both qualitative and quantitative approaches were used:

- Qualitative Content Validity

In the qualitative approach, a group of 10 experts evaluated the appropriateness of language, the placement of items, and the appropriateness of scoring for each item. The suggested modifications by the experts in the research team were reviewed and applied.

- Quantitative Content Validity

For quantitative content validity assessment, the Content Validity Ratio (CVR) and Content Validity Index (CVI) for individual items were calculated [33]:

Content Validity Ratio (CVR):

The scale was provided to eight experts, and they were asked to rate each item as essential, useful but not essential, or not essential. Then, the CVR was calculated. A CVR value greater than 0.75 for 8 experts indicated the necessity and importance of the item in the scale [33].

Content Validity Index (CVI):

The scale was provided to 10 experts, and they were asked to rate the relevance of each item on a four-point scale (one = not relevant, two = somewhat relevant, three = relevant, 4 = very relevant). The CVI was calculated by dividing the number of experts who rated an item as 3 or 4 by the total number of experts. Then the modified kappa were calculated. Kappa greater than 0.75 were considered excellent [35].

- Item Analysis

In this study, correlation between each item and the total score of the scale was calculated for a sample of 30 family caregivers of older adult patients undergoing hemodialysis. Items with a correlation coefficient less than 0.30 with the total score of the test were considered for elimination [36].

- Structural Validity

To assess the structural validity, Exploratory Factor Analysis (EFA) and Confirmatory Factor Analysis (CFA) were employed. Four hundred older adult patient caregivers undergoing hemodialysis completed the scale. The sample was randomly split into two samples of 200 each for EFA and CFA.

- EFA

EFA was performed using the maximum likelihood method by SPSS version 24. Factor retention was determined based on eigenvalues greater than 1 and scree plot [37]. Only factors with eigenvalues equal to or greater than 1 were considered significant [33, 36, 38]. The critical value for factor retention was set at 0.3 [39]. Before conducting EFA, skewness less than ± 3 and kurtosis less than ± 7, the absence of outliers based on the box plot, and the presence of correlations between 0.30 and 0.70 among the items were checked. The factor recommended a minimum of three items. The Bartlett’s test (should be significant) and the Kaiser-Meyer-Olkin (KMO) (greater than 0.70) were used to assess the adequacy of the sample. Additionally, items with communalities of less than 0.20 were removed [40, 41].

- CFA

CFA was conducted with the second sample of 200 participants using the AMOS software version 26. Fit indices including CFI, GFI and TLI (> 0.9), PCFI and PNFI (> 0.5), RMSEA (< 0.08), and CMIN/DF were examined to assess model fit [33].

- Reliability

The internal consistency of the Caregiver Preparedness Scale was evaluated by computing Cronbach’s alpha and McDonald’s omega coefficient (both should be at least 0.70), as well as the Average Inter-item Correlation (AIC) which should fall between 0.2 and 0.4 [39, 42, 43].

### Findings

#### Demographics

Out of the 400 caregivers who participated in the study, 202 (50.5%) were female. The mean and standard deviation of age of the samples was 40.93 ± 12. Additional demographic findings are shown in Table 1.

**Table 1:** Demographic characteristics of family caregivers of the older adults undergoing hemodialysis (*N* = 400)

Demographic variables		Number (%)/ Mean (SD)^*^
Caregiver`s Gender	Female Male	202 (50.5) 198 (49.5)
Caregiver`s Education level	Under diploma Diploma Associate Degree Bachelor of Science Master and higher	98 (24.5) 113 (28.5) 21 (5.3) 136 (34) 32 (8)
Relationship to the patient	Patient’s child Spouse Patient’s brother Patient’s sister other	208 (52) 74 (18.8) 17(4.3) 19 (4.8) 82 (20.5)
Patient`s Gender	Male Female	208(52.3) 191(47.8)
Patient`s marital status	Married Single Others	174(87) 9(4.5) 17(8.5)
Dialysis sessions per week	three times twice once Four times	99(49.5) 73(36.5) 22 (11) 6 (3)
Caregivers age	40.93 (12) *	
Patients age	69.22 (6.98) *	
Duration of hemodialysis treatment (Year)	3.66 (2.72) *	

* Mean and standard deviation are provided.

#### Translation

During the translation and cultural adaptation process, the term “your family member” was replaced with “your patient,” according to the translators, expert panel, the scale designer, and the research team. In items 1, 2, and 6, the phrase “how much” was removed from the beginning of the sentence and placed before the verb at the end of the sentence to match the semantic meaning with the questioning style in Iranian culture. Furthermore, to eliminate ambiguity in item 3, the word “centers” was added to “service providers,” resulting in “service providers and centers.” In item 4, the word “coping with stress” was used instead of “tolerating stress.”

### Psychometric evaluation of the caregiver readiness scale

Quantitative Face Validity:

All items had an impact score greater than 1.5. Therefore, no items were deleted.

Content Validity:

Experts provided feedback on language, appropriate wording, proper placement of items, and appropriate scoring. The CVR and Kappa coefficient of all items were higher than 0.75. Therefore, no items were deleted. The scale content validity index was calculated using the average method and resulted in a score of 1, which is above 0.90 and considered acceptable.

Item Analysis:

Cronbach’s alpha and standardized Cronbach’s alpha were equal 0.95 and 0.95, respectively. One item (item #2) had a correlation of less than 0.30 with the total score and was deleted based on the research team’s decision (Table 2).

**Table 2:** Results of the caregiver preparedness scale item analysis

Number	Items	Corrected Item-Total correlation	Cronbach’s Alpha if item deleted
1	How well prepared do you think you are to take care of your patient’s physical conditions?	0.493	0.786
2	How well prepared do you think you are to meet your patient’s emotional needs?	0.189	0.821
3	How well prepared do you think you are to cope with the stress of caring for your patient?	0.541	0.775
4	How well prepared do you think you are to make caring activities enjoyable for you and your patient?	0.664	0.752
5	How well prepared do you think you are to become aware of the service centers and their services and provide them to your patient?	0.697	0.750
6	How well prepared do you think you are to respond to and manage emergencies that happen to your patient?	0.565	0.771
7	How well prepared do you think you are to get the help and information you need from the healthcare system?	0.403	0.793
8	In general, How well prepared do you think you are to take care of your patient?	0.727	0.767

Structural Validity:

The KMO was 0.914, and Bartlett’s test of sphericity was significant at *p* < 0.001. No items were missing data, and there were no outliers based on the box plot. All items had skewness less than 3 and kurtosis less than 7, confirming the absence of significant deviations from normality. Items had correlations 0.30 to 0.70 with each other.

**Fig. 1:**
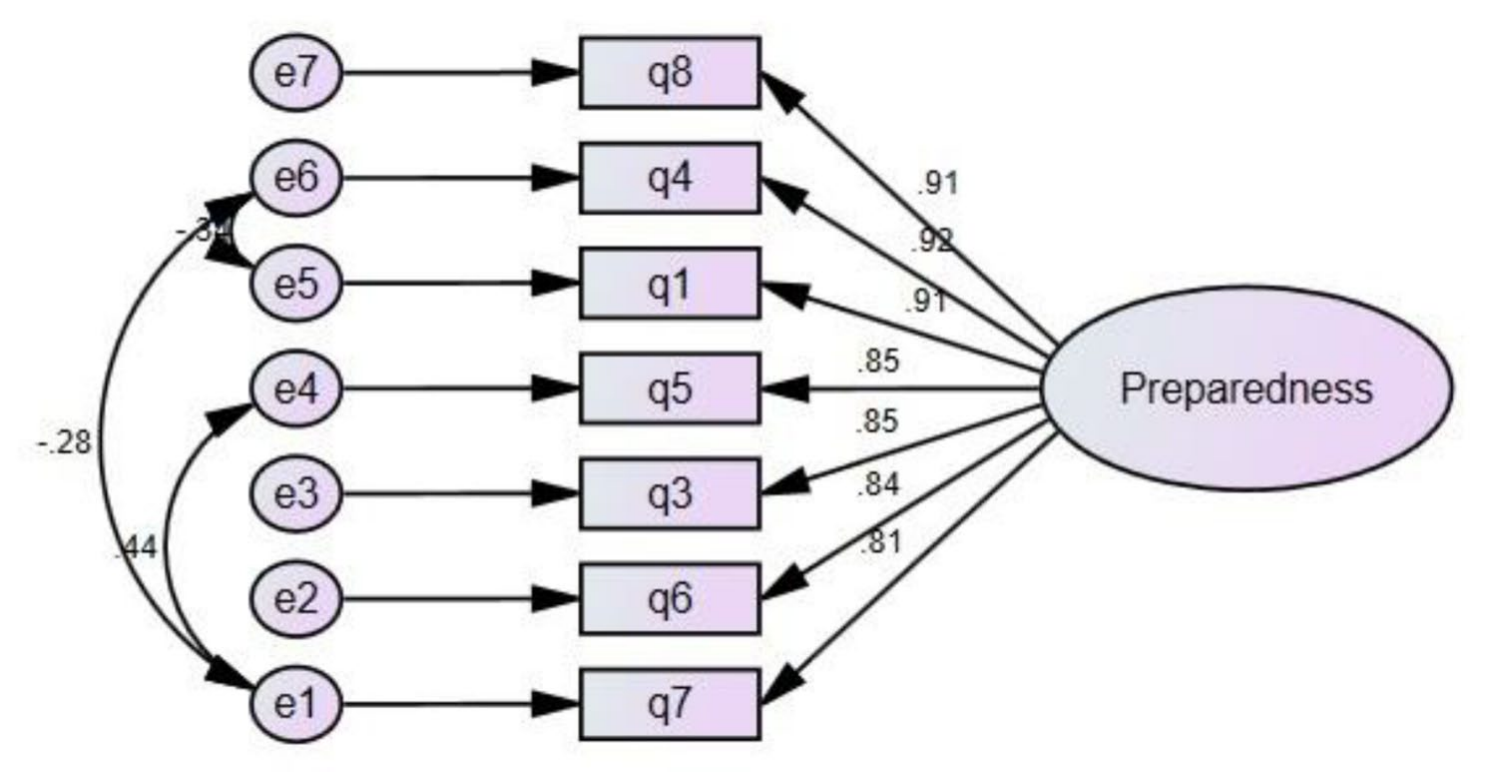
The final structure model of the caregiver preparedness scale

The scale with seven items underwent EFA, and based on the results, only one factor composed of all the items was extracted, explaining 75.7% of the total variance (Table 3). CFA was performed after confirming the assumptions, and model fit indices were examined. With model modification (three measurement error covariance between items 5 and 7, 4 and 7, 1 and 4), model fit indices were calculated, indicating an acceptable fit for a single-factor structure (Table 4) (Fig. 1).

**Table 3:** Results of exploratory factor analysis of the caregiver preparedness scale (*N* = 200)

Factors	Items	Factor loading	h^2^	λ	% Variance
1	8- In general, How well prepared do you think you are to take care of your patient?	0.912	0.832	5.3005	75.7
	4- How well prepared do you think you are to make caring activities enjoyable for you and your patient?	0.892	0.796		
	1- How well prepared do you think you are to take care of your patient’s physical conditions?	0.892	0.795		
	5- How well prepared do you think you are to become aware of the service centers and their services and provide them to your patient?	0.868	0.753		
	3- How well prepared do you think you are to cope with the stress of caring for your patient?	0.860	0.739		
	6- How well prepared do you think you are to respond to and manage emergencies that happen to your patient?	0.836	0.699		
	7- How well prepared do you think you are to get the help and information you need from the healthcare system?	0.828	0.685		

**Abbreviations**: h2: Communalities, ʎ: Eigenvalue

**Table 4:** The fit model indices of confirmatory factor analysis of the caregiver preparedness scale

Chi-Square, df, *P*-value	CMIN/DF	RMSEA	TLI	PCFI	GFI	CFI	PNFI
24.13,11,*p* < 0.01	2.194	0.077	0.982	0.519	0.969	0.991	0.515

Reliability:

The Cronbach’s alpha and Omega coefficients of 0.956 were obtained. The AIC was 0.756. The final Farsi-version of the caregiver preparedness scale is shown in Table 5.

**Table 5:** The final Farsi-version of the Caregiver Preparedness Scale

*N*	Items	Not at all prepared	Not too well prepared	Somewhat well prepared	Pretty well prepared	Very well prepared
1	In general, How well prepared do you think you are to take care of your patient?					
2	How well prepared do you think you are to make caring activities enjoyable for you and your patient?					
3	How well prepared do you think you are to take care of your patient’s physical conditions?					
4	How well prepared do you think you are to become aware of the service centers and their services and provide them to your patient?					
5	How well prepared do you think you are to cope with the stress of caring for your patient?					
6	How well prepared do you think you are to respond to and manage emergencies that happen to your patient?					
7	How well prepared do you think you are to get the help and information you need from the healthcare system?					

## Discussion

Preparedness to predict potential problems and find possible solutions for them has been proposed as an ability, which requires the development of capabilities and skills. Measuring preparedness provides valuable information about individuals’ ability to behave appropriately in different situations. The Caregiver Preparedness Scale has been translated and validated in various languages and cultures globally, but it has not been translated and validated in Farsi language so far. Therefore, this study aimed to translate and validate this scale for family caregivers in Iranian communities. The study results showed that the Farsi version of this scale has good validity and reliability.

In this study, some modifications were done especially on items number 3 and 4. In this context, the study by Gutierrez-Baena and Romero‐Grimaldi in (2021) states that in the field-test of the Spanish version of the scale, caregivers did not have a correct understanding of item 5. They also concluded that items 3 and 5 have similar meanings and that a social aspect should be added to item 7, rather than just asking about the healthcare system. Furthermore, based on qualitative study results, they believed that spiritual needs of the patient should also be questioned. Therefore, instead of removing item 3, they added a new item to the scale. They also made some modifications to item 5 [27].

In the item analysis phase, item 2, titled “How prepared do you think to meet your patient’s emotional needs?” was removed according to the research team’s opinion. It appears that in this study, the concept of preparedness from the perspective of caregivers of hemodialysis patients was primarily related to physical aspects of care. Moreover, the complex physical challenges faced by patients undergoing hemodialysis, coupled with the multitude of physical care tasks that caregivers must be prepared for, may have overshadowed the importance of preparedness to address the emotional needs of the patient among these caregivers. Therefore, based on the caregivers’ responses in the present study, they did not perceive preparedness for psychological care as distinct from preparedness for caregiving.

In this study, following EFA, only one factor composed of all the items was extracted, indicating that the Caregiver Preparedness Scale is a unidimensional measure. In the original study by Archbold et al., (1990), the scale was also introduced as a unidimensional scale [22]. The unidimensionality of the scale in most studies in other populations has been confirmed, too [22, 31, 44–46]. Gutierrez-Baena and Romero‐Grimaldi (2020) also obtained a one-factor structure through EFA, explaining 59% of the variance [27]. Ugur et al., (2017) identified one factor using principal component analysis, explaining 56% of the variance [47]. The variance explained by the Farsi version of the scale with 7 items was higher than in other studies [22, 27, 31, 45, 47], which is a strength of this version and the current study.

Also, in line with findings of this study, Kuzmik et al., (2021) [46], Petruzzo et al., (2017) [44], Pucciarelli et al., (2014) [31], and Henriksson et al., (2012) [45], through confirmatory factor analysis, have also confirmed the primary one-factor structure of the scale.

The findings of this study indicated high internal consistency of the scale. Cronbach’s alpha in the psychometric study of the Gutierrez-Baena B, Romero‐Grimaldi., was 0.89 [27], also, in a sample of caregivers of heart failure patients and stroke survivors, it was 0.91 and 0.94, respectively [31, 44].

This research also had its limitations. The study did not assess the test-retest reliability, and other psychometric indices such as standard error of measurement and responsiveness were not investigated.

## Conclusion

In conclusion, the findings of the present study demonstrate that the Caregiver Preparedness Scale provides acceptable psychometric. The use of the Caregiver Preparedness Scale may assist healthcare providers in identifying family members with lower preparedness for caregiving and in assessing specific areas that require interventions. Increased support for family caregivers with lower preparedness may help them enhance their readiness for caregiving, allowing caregivers to better align with their caregiving role. Suggestions are made for future research to examine the scale in other psychometric parameters and to evaluate its use and validity in other family caregiver populations.

## Data Availability

The datasets used and analyzed during the current study are available from the corresponding author on reasonable request.

## Abbreviations

CVR: Content Validity Ratio
CVI: Content Validity Index
EFA: Exploratory Factor Analysis
CFA: Confirmatory Factor Analysis
KMO: Kaiser–Meyer–Olkin
CFI: Comparative Fit Index
PNFI: Parsimonious Normed Fit Index
GFI: Goodness-of-Fit Index
PCFI: Parsimonious Comparative Fit Index
TLI: Tucker-Lewis Index
RMSEA: Root Mean Square Error of Approximation
AIC: average inter-item correlation
